# Influence of Exercise on Exhausted and Senescent T Cells: A Systematic Review

**DOI:** 10.3389/fphys.2021.668327

**Published:** 2021-08-20

**Authors:** Thomasina Donovan, Amanda L. Bain, Wenjuan Tu, David B. Pyne, Sudha Rao

**Affiliations:** ^1^Research Institute for Sport and Exercise, University of Canberra, Canberra, ACT, Australia; ^2^Australian Centre for Health Services Innovation and Centre for Healthcare Transformation, School of Public Health and Social Work, Faculty of Health, Queensland University of Technology, Brisbane, QLD, Australia; ^3^Gene Regulation and Translational Medicine Laboratory, Immunology Department, QIMR Berghofer Medical Research Institute, Brisbane, QLD, Australia

**Keywords:** exhausted, senescent, T cells, immune cells, exercise, fitness

## Abstract

The impaired effector function of exhausted and senescent T cells is implicated in cancer progression and inadequate vaccine responses. Exercise has been shown to improve cancer therapy and vaccine efficacy, most likely by improving immune function. However, given inconsistent terminology and definitions, the interactions between exercise and exhausted and senescent T cells remain unclear. We therefore performed a systematic review to investigate the effect of exercise on senescent and exhausted CD8^+^ T cell populations clearly defined by protein surface markers. Thirty articles were included, with the majority (*n* = 24) reporting senescent T cell populations defined according to a variety of surface markers. Repeated exercise was shown to be beneficial through limiting the accumulation of senescent and exhausted CD8^+^ T cells. This outcome is likely related to exercise-induced preferential mobilization of senescent T cells promoting apoptosis in the peripheral blood compartment. Future studies need to determine the clinical relevance of this effect in cancer prevention and vaccine efficacy. Data regarding exercise and exhausted T cells are limited due to a lack of available high-quality studies. Future studies require the control of confounding variables such as sex and cytomegalovirus (CMV) status, and consistent definitions of exhausted and senescent T cell populations to improve comparisons between studies and interventions.

## Introduction

As drivers of the adaptive immune response, CD8^+^ T cells are important actors in cancer prevention and vaccine efficiency. However, exhausted or senescent CD8^+^ T cells have impaired effector function, thereby contributing to cancer development and decreasing vaccine efficiency (McElhaney et al., 2016; Huff et al., 2019). Exhausted and senescent T cells are present in the tumor microenvironment and in older adults, who typically have impaired vaccine responses (Simpson et al., 2012; Crespo et al., 2013). The terminology and definitions for senescent and exhausted CD8^+^ T cells are yet to be finalized resulting in a lack of clarity in research for these cell types. In the past, senescent cells have been referred to as late-stage, terminally, or highly differentiated T cells (Turner, 2016; Duggal et al., 2019). Adding to the confusion, the terms “exhausted” and “senescent” T cells have been used interchangeably (Bigley et al., 2013; Pawelec, 2019). Exhausted and senescent T cells have different origins, identifications (protein surface markers), and functional ability. Exhausted T cells arise from excessive and prolonged stimulation of the T cell receptor (TCR) and the action of inflammatory cytokines, which progressively suppress T cell effector functions (Pawelec, 2019). In contrast, senescent T cells arise through aging and/or chronic infection (Turner, 2016). The two cell types can be distinguished by protein surface marker expression and other features, as outlined in Table 1 (Crespo et al., 2013; Huff et al., 2019). Although different cell types, exhausted and senescent CD8^+^ T cells share similarities. For example, cytomegalovirus (CMV) infection can drive the development of both cell types, KLRG1^+^ is a common surface marker, and both subtypes have a reduced ability to produce cytokines (Simpson et al., 2016; Huff et al., 2019). Some researchers propose there are benefits to senescent T cells such that they maintain their functionality as anti-CMV immunosurveillance by being prevented from further expansion, and lost by clonal attrition (Pawelec, 2019). Similarly, exhausted T cells could be advantageous in autoimmune disease and organ transplantation (Pawelec, 2019). However, regarding cancer and vaccine efficiency evidence indicates that senescent and exhausted T cells are a burden (McElhaney et al., 2016; Huff et al., 2019).

**Table 1:** Typical surface markers and established characteristics of exhausted and senescent CD8^+^ T cells.

**CD8^**+**^** **cell type**	**Surface** **markers**	**Other** **characteristics**
Exhausted	PD-1^+^ CTLA-4^+^ TIM3^+^ LAG3^+^ KLRG1^+^	• Impaired cytokine expression—IFN-γ, TNF-α, IL-2 • Decreased cytolytic ability • Decreased proliferative capacity • Sustained upregulation and expression of surface inhibitory receptors (listed to the left)
Senescent	CD28^−^ CD57^+^ KLRG1^+^ CD27^−^	• Shortened telomeres • Cell cycle arrest • Altered sensitivity to apoptosis • DNA damage • Defective mitochondrial function • Higher p53

Given the pathophysiological consequences of T cell senescence and exhaustion, efforts are underway to overcome impaired exhausted and senescent T cell function. One approach has been to exploit epigenetic mechanisms to improve immunotherapy and vaccine efficiency (Dan et al., 2020). Epigenetics is the study of potentially heritable changes in gene expression without changes in the DNA base sequence (Berger et al., 2009), and these dynamic mechanisms can be influenced by many environmental factors (Ling and Ronn, 2014). One important environmental epigenetic modifier is exercise, and recognition of its importance in influencing outcomes is reflected in its prescription for treatment of at least 26 different diseases or conditions, including psychiatric, neurological, metabolic, cardiovascular, pulmonary, musculoskeletal, and cancer (Pedersen and Saltin, 2015). There has been little research on the effect of exercise on exhausted T cells, with greater focus on its effect on senescent T cells. In their 2012 review, Simpson et al. (2012) reported that many cross-sectional studies demonstrate that exercise reduces the numbers of senescent T cells and increases T cell proliferation. However, there is conflicting evidence on the extent to which exercise influences T cell proliferation, most likely related to methodological variations in both the exercise interventions and definitions of senescent T cell populations in different studies (Simpson et al., 2012). The definition of senescent (and exhausted) T cells has been refined in recent years, and using the current definitions presented in Table 1, uniform studies could be designed to define the impact of exercise on these populations (Huff et al., 2019). Studying epigenetics in the clinic, laboratory, and field should be useful for understanding how exercise influences these cells, the effectiveness of various interventions, and ultimately clinical outcomes.

The aim of this systematic review was to aggregate current knowledge on the effect of exercise on exhausted and senescent CD8^+^ T cells, which are implicated in cancer progression and vaccine responses. We sought to differentiate the effects of acute (single bouts) exercise with the cumulative effects of repeated exercise on these types of T cells. In doing so, we identify areas requiring further investigation, optimal experimental approaches for these investigations, and how rigorous definition of cell populations and outcomes may promote exercise as an adjunct therapy for cancer and vaccines by limiting exhausted and senescent T cell numbers.

## Methods of Systematic Review

### Protocol

The systematic review followed the Preferred Reporting Items for Systematic Reviews and Meta-Analyses (PRISMA) guidelines for systemic reviews (Moher et al., 2009).

### Search Strategy

The electronic databases of PubMed, Embase, and Web of science were searched between January 2010 and 11 April 2021 (last date searched). Subject headings and keywords such as “T cell,” “senescence,” “exhaustion,” specific biomarkers, and “exercise” were combined in the search. The detailed search strategy is outlined in Supplementary Table 1.

### Eligibility Criteria

The PICOS framework was used to define the eligibility criteria. Studies that met the following criteria were included: (1) *P* (population): adults (over 16 years old), *in vivo* human studies, no restriction on sex or physical activity level; (2) *I* (intervention/exposure): acute and repeated exercise, physical activity level, or sport; (3) *C* (comparison): control group or baseline data; (4) *O* (outcomes): reported on the number/proportion of a protein-surface-marker-defined senescent or exhausted CD8^+^ T cell population/s; (5) *S* (study designs): cross-sectional, longitudinal, intervention, randomized controlled trial (RCT), or randomized cross-over trial (RCOT). Studies with the following characteristics were excluded: animal studies, reviews, abstracts only, case studies, commentary pieces, non-English studies, or studies investigating participants under the age of 16 years old, have immune system comorbidities, have contraindications to exercise therapy, or are pregnant. If more than one article reported on the same cohort population, only one study was included based on relevance and recency.

### Study Selection

Article titles and abstracts were first screened, followed by screening of the full text to confirm that the study fit the eligibility criteria. Two authors (TD and DP) independently conducted the entire screening process, and discrepancies were resolved through discussion.

### Data Collection

The first author, year published, study design, defined exhausted or senescent T cell population/s, cohort description, exercise intervention (if applicable), data collection, and results on the impact of exercise on the exhausted or senescent T cell population/s were collated and presented.

### Quality Assessment

National Institutes of Health (NIH) quality assessment (QA) tools were employed to evaluate the included studies (National Heart Lung and Blood Institute, 2021). Specifically, the NIH QA tool for observational cohort and cross-sectional studies, and the NIH QA tool for a before-after (pre-post) study with no control group, were adapted to create a single QA tool (see Supplementary Table 2) as several included studies featured both cross-sectional and intervention arms. In addition, RCT and RCOT studies were assessed using the NIH QA tool for controlled intervention studies (see Supplementary Table 3). All QA tool outcomes were identical to permit comparison of different study types, and assessment of quality was determined as either poor, fair, or good.

## Results

### Overview of Included Studies

**Figure 1:**
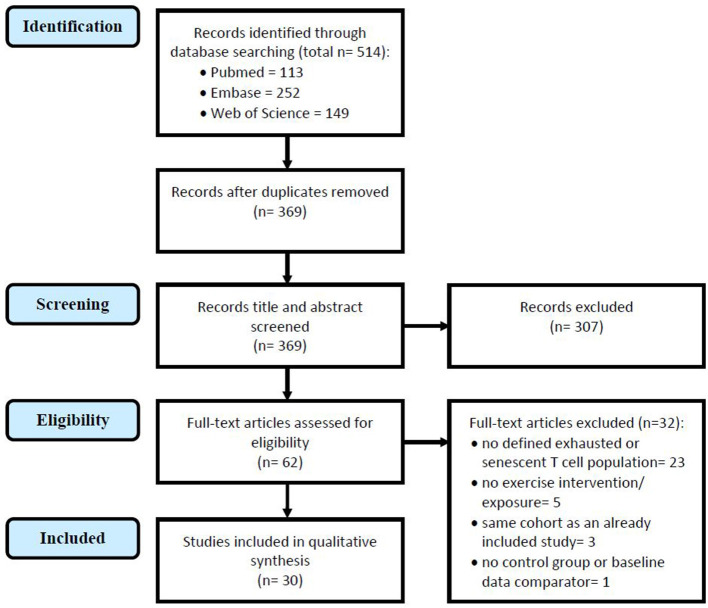
Study selection in accordance with the Preferred Reporting Items for Systematic Reviews and Meta-Analyses (PRISMA) guidelines for systemic reviews (Moher et al., 2009), the above flowchart illustrates the selection process of studies in this review.

The search yielded 369 articles, without duplicates. Titles and abstracts were screened for suitability which eliminated 307 ineligible articles, the majority of which were reviews and conference abstracts. The full text of 62 studies were screened to assess their eligibility, and 32 studies were eliminated due to the following reasons: not investigating a senescent or exhausted CD8^+^ T cell population defined by protein surface markers (*n* = 23); no exercise intervention or exposure level was studied (*n* = 5); participant cohort was the same as an already included study (*n* = 3); or no control group or baseline data was reported (*n* = 1). Figure 1 illustrates the study selection process. A final total of 30 included studies investigated the impact of exercise on senescent (*n* = 24) and/or exhausted (*n* = 7) CD8^+^ T cells—one study investigated both populations.

The type of study, protein surface marker/s used to define the CD8^+^ T cell population, cohort characteristics, data collection methods, exercise intervention details, and outcomes of the included studies are outlined in Table 2 (senescent T cells) and Table 3 (exhausted T cells). Briefly, the majority of included studies had features of cross-sectional and/or intervention analyses. Various protein surface markers were used to identify senescent and exhausted CD8^+^T cell populations, it is beyond the scope of this review to determine the optimal/most valid biomarker combination. The most commonly reported cohort characteristics were age, physical activity level, and sex. All included studies collected venous blood samples to investigate their aims and most (*n* = 15) reported VO_2_max (maximal aerobic capacity) scores as a measure of aerobic fitness. Acute exercise was more commonly investigated (*n* = 21) than repeated exercise completed over multiple days and/or weeks (*n* = 10). The types of acute exercise examined included: cycling (*n* = 14), running (*n* = 6), futsal game (*n* = 1), strength (*n* = 1), and swimming (*n* = 1). Resistance training (*n* = 3), cycling (*n* = 2), walking (*n* = 2), running (*n* = 2), swimming (*n* = 1), and triathlon training (*n* = 1) were studied as types of repeated exercise.

**Table 2:** Summary of results detailing the influence of exercise on senescent T (Ts) cells from studies included in this systematic review.

**Reference** **Study design** **CD8+ Ts population**	**Cohort**	**Data collection**	**Exercise intervention**	**Outcome**
Bigley et al., 2012 I KLRG1+	*n* = 20 healthy males, 22–35 years. **Excluded:** smokers, medications/supplements, infection in previous 6 weeks.	• Blood samples: at rest, immediately after, and 1 h post. • VO_2_max	Acute Exercise—cycling. 30 min cycling at 85% VO_2_max. Submaximal cycling protocol to determine VO_2_max.	1. Exercise mobilized Ts.
Ingram et al., 2015 I KLRG1+	*n* = 10 males, 27 years. **Included:** non-smokers, taking no medication/vitamins, familiar with cycling time trials (TT), free from illness 2 weeks prior.	• Blood samples: at rest, immediately after, and 1 h post. • HR, RPE, sleep behavior	Acute Exercise—cycling. 1 h cycling at 90% wattage—determined from 40 km cycle TT.	1. Exercise mobilized Ts. Values fell below pre levels at 1 h post exercise (cells/μl). • At rest = 170 • Immediately post = 220–300 • 1 h post = 100
Karim and Jabbar, 2018 I KLRG1+	Elite male athletes, 26 years, normobaric only: • Normobaric Control (NC), *n* = 11. • Normobaric Exercise (NE), *n* = 12. ** Included:** healthy, non-smokers, non-medication/vitamin users, not experienced altitude >3,000 m in 12 months prior.	• Blood samples: at rest, immediately after, and 2 h post GXT– at baseline and post exercise. • VO_2_max • HRRmax	Repeated and Acute Exercise—running. • NC = sedentarily experienced normobaric conditions. • NE = training treadmill for 45 min at 30–65% HRRmax, 5 days/week, for 4 weeks. GXT = incremental treadmill protocol to determine VO_2_max.	1. GXT mobilized Ts in both NC and NE. Values fell below resting values 2 h post.
Minuzzi et al., 2018 I/C KLRG1+	Master athletes, >20 years training/competition and currently participating regularly. *n* = 19 (15 males, 4 females), 40–60 years. Control group, weight and aged matched, performed no regular training in last 20 years. *n* = 10 (7 males, 3 females) control group, 40–60 years.	• Blood samples: at rest, 10 min post, and 1 h post exercise. • VO_2_max • HR, RPE	Acute Exercise—cycling. Incremental cycling protocol to determine VO_2_max.	1. Control Ts **>** Masters Ts. 2. Time of measurement controlled, master athletes still had ↓ Ts, ~13.1%. 3. Exercise mobilizes Ts.
Wang et al., 2011 I CD28– (Ts_1_) and KLRG1+ (Ts_2_)	*n* = 50 males, 20–25 years, normoxic only: • **N–C** = normoxic control • **N–T** = normoxic exercise ** Included:** non-smokers, non-med/vitamins, infection/cardio risk-free, no regular PA or high altitude <1 year.	• Blood samples: at rest, immediately, and 2 h after graded exercise test (GXT). • Daily physical activity questionnaire.	Repeated Exercise—cycling. 30 min/day, 5 days/week for 4 weeks of either: • N–C = 21% O_2_ at rest • N–T = 50%Wmax in 21% O_2_ 48 h before/after intervention GXT (bicycle exhaustion protocol)	1. Both Ts unchanged after interventions. 2. Exercise mobilizes Ts.
Bastos et al., 2020 C CD28–	*n* = 53 healthy males, 65–85 years, grouped based on physical fitness: - low (*n* = 23) - moderate = 2–3 × /week (*n* = 7) - high: (*n* = 23) ** Excluded:** immune system co-morbidities, immunosuppressive drugs, smoking, alcohol abuse.	• Blood sample at rest. • VO_2_max	No exercise intervention. Treadmill test to determine VO_2_max.	1. No differences in % Ts among groups.
Cury-Boaventura et al., 2018 I CD28–	*n* = 16 males, 26 years. **Included:** professional futsal athletes for at least 10 years, 4 h futsal training ×5/week. **Excluded:** history of infection, viruses, chronic lesions, diabetes, rheumatoid arthritis, hormonal dysfunction, lupus, or other inflammatory or hematological diseases, taking medication.	• Blood samples: pre and post exercise.	Acute Exercise—futsal game. 2 × (5 min futsal + 5 min recovery).	1. After exercise, CD28 expression ↓1.9-fold—thus cells are presenting more Ts.
Ross et al., 2018 I/C CD28–	*n* = 9 **younger** males, 18–25 years. *n* = 10 **older** males, 60–75 years. **Included:** Physically active (moderate intensity physical activity >2/week), BMI <30, non-smokers, no medication, no CVD/diabetes.	• Blood samples: at rest, immediately after, and 1 h post exercise. • VO_2_peak • Height, BM, BP	Acute Exercise—cycling. 30 min cycling at 70% VO_2_peak. Incremental graded exercise test to exhaustion determined VO_2_peak.	1. Older Ts cells (~55%) > Younger Ts cells (~30%). 2. Exercise mobilizes Ts, regardless of age. 3. Older group, greater ingress, and egress of Ts than CD28+/CD8+. No difference within young group.
Shimizu et al., 2011 I CD28–	*n* = 24 males and females, 67 years. • Exercise training group (EXC), *n* = 12 • Non-exercise control group (CON), *n* = 12 ** Included:** healthy, sedentary, elderly, living independently, passed medical examination in last year, received permission from sports doctor. ** Excluded:** hormone replacements, infection in last 3 months, metabolic disorders, and major surgery in last 6 months.	• Blood samples: pre and post exercise. • Strength test—week 0, 4, 8, 12.	Repeated Exercise—resistance training. EXC: resistance training for 2 days/week for 12-week. 20, 30, 40% 1RM at week 1, 3, 5, respectively. CON: no formal exercise outside usual daily activities.	1. EXC ↑# CD28+/CD8+ cells (100–125 cells/μl). 2. CON showed no significant change in CD28+/CD8+ #.
Silva et al., 2016 C CD28–	46 males, 65–85 years, from community-based exercise programs categorized into: • never trained, *n* = 15 • moderately trained (2–3/week), *n* = 16 • intense trained (≥5days/week, regularly for past 5 years), *n* = 15 ** Excluded:** smoking, alcohol abuse, immune system comorbidities, immunosuppressive drugs.	• Blood sample at rest. • VO_2_max • IPAQ	No exercise intervention.	1. No difference in Ts proportions across groups: • Never trained = 52.6% • Moderately trained = 50.7% • Intense trained = 57.1%.
Brown et al., 2014 I/C CD28–/CD57+	Trained (well-trained soccer players). • *n* = 8, males, 18 years. • *n* = 8, females, 19 years. Untrained (exercising less than UK Health Dept. recommendation of 2–3 h/week). • *n* = 8, males, 19 years. • *n* = 8, females, 19 years. ** Included:** non-smokers, no medications, infection-free for 6 weeks prior.	Blood samples: at rest, immediately after, and 1 h post exercise.	Acute Exercise—running. Incremental treadmill test to exhaustion.	1. ~45% ↑ Ts immediately post exercise and ~51% ↓ 1 h post exercise. 2. Untrained Ts proportions > trained, across all time points (rest, immediately after, and 1 h post): • Female: 21 > 17, 34 > 26, 13 > 13 • Male: 34 > 31, 54 > 36, 26 > 23
Brown et al., 2015 I/C CD28–/CD57+	Trained (well-trained soccer players). *n* = 13, females, 20 years. Untrained (exercising <150 min of deliberate moderate to vigorous per week). *n* = 13, females, 20 years. ** Included:** non-smokers, no medications, infection-free for 6 weeks prior.	• Blood samples: pre and post exercise. • HR, IPAQ, blood lactate.	Repeated and Acute Exercise—running + strength. Trained = 20 sessions over 2-week training camp: field-based training (*n* = 12) + pool based recovery (*n* = 2) + competitive matches (*n* = 2) + resistance exercise (*n* = 4). Untrained = physical activity monitored for 2 weeks Incremental treadmill test to exhaustion.	1. No difference in Ts #/% between trained and untrained. • Trained i. Pre = 70 ± 46 cells/μl ii. Post = 75 ± 73 cells/μl • Untrained i. Pre = 59 ± 47 cells/μl ii. Post = 71 ± 69 cells/μl
Cao Dinh et al., 2019 RCT CD28–/CD57+	Total of 100 females, 65–75 years, split among: • intensive strength training (**IST**), *n* = 31 • strength endurance training (**SET**), *n* = 33 control (**CON**), *n* = 36 ** Excluded:** performing physical exercise currently or within past 6 months, regularly at higher intensities than habitual daily activity.	• Blood samples: before and after 6-week intervention.	Repeated Exercise—resistance training. 6 weeks resistance training, 2–3x/week of either; IST = 3 ST10 repetitions at 80% 1RM; SET = 2 ET30 repetitions at 40% 1RM; or CON = flexibility training	1. SET ↓~24% Ts proportion, in CMV+. Effect is significant compared to CON. 2. Ts unchanged in IST or CON.
Duggal et al., 2018 C CD28–/CD57+	Amateur non-elite cyclists= cycled 100/60 km <6.5/5.5 h twice in 3 weeks prior to testing; and maintained most of adult life. *n* = 125 (84 males, 41 females), 55–80 years. Controls Old = (55–80 years) did not partake in regular PA. *n* = 75 (43 males, 31 females). • Young = (20–35 years) not involved in regular intense exercise. *n* = 55 (30 males, 25 females).	• Blood sample at rest.	Repeated Exercise—cycling.	1. Older adults and master cyclists Ts > young Ts. 2. Older adults Ts ≈ master cyclists Ts
Krüger et al., 2016 I CD28–/CD57+	*n* = 32 untrained males, 26 years. All completed both HIT + CONT, 7 days apart. • **Included:** no participation in competitive sports, <3 h regular sport activities/week, <55 ml/min/kg VO_2_max, good physical health.	• Blood samples: at rest, immediately after, 3 h post, and 24 h post exercise. • VO_2_max	Acute Exercise—cycling. HIT = 5 × 3 min at 90% peak power output cycling. CONT = cycle at 70% VO_2_max for 30 min. Incremental cycling protocol to determine VO_2_max.	1. Both HIT and CONT mobilized Ts, HIT > CONT.
Curran et al., 2019 I CD28–/CD27–	*n* = 12 males, 16–65 years, control (CON) only.	• Blood samples: at rest, immediately, and 1 h post exercise. • VO_2_max • IPAQ	Acute Exercise—cycling. 30 min cycling at 80% VO_2_max. Incremental submaximal cycling test to determine VO_2_max.	1. Vigorous acute exercise mobilized Ts. • At rest = 33 cells/μl • Immed. Post = 55 cells/μl • 1 h post = 22 cells/μl
LaVoy et al. (2017) I CD28–/CD27–	*n* = 17 males and females cyclist, 31 years. **Included:** participation in road cycling at least 3 × a week for 12 months. **Excluded:** smokers and use of immune-modulatory medications.	• Blood samples: at rest, immediately, and 1 h post exercise. • HR, RPE, lactate threshold (LT)	Acute Exercise—cycling. 30 min cycling at −5, +5, and +15% LT.	1. Intervention mobilized Ts. Values returned to or below baseline. E.g., in +5% LT, CMV+: • At rest = 36 cells/μl • Immed. Post= 71 cells/μl • 1h post= 29 cells/μl 2. ↑↑ intensity, ↑Ts mobilization.
Turner et al., 2010 I CD28–/CD27–	*n* = 14 males, 35 years. **Included:** healthy, accustomed to vigorous endurance exercise, VO_2_max in 90th percentile for their age. **Excluded:** smoking, use of vitamins in the last 6 weeks	• Blood samples: at rest, immediately, and 1 h post exercise. • VO_2_max • IPAQ	Acute Exercise—running. EXC = 60 min treadmill running at 80% of VO_2_max. CON = 2 h sitting in same room. Incremental treadmill-running protocol to determine VO_2_max.	1. Intervention mobilized Ts, and at a greater rate than other phenotypes: • Late (Ts) = +265, −60% • Intermediate= +154, −52% • Early= +67, −28%
Lavoy et al., 2014 I CD28–/KLRG1+	*n* = 32 males, 39 years. **Included:** physically active, non-smoker	• Blood samples: at rest, immediately, and 1 h post exercise. • VO_2_max • HR	Acute Exercise—cycling. 30 min cycling at 80–85% VO_2_max. Incremental submaximal cycling test to determine VO_2_max.	1. Intervention mobilized Ts. Values fell below resting values 1 h post. E.g., in HSV+/CMV+: • At rest = 119 cells/μl • Immed. Post = 422 cells/μl • 1h post = 79 cells/μl
Spielmann et al., 2014 I/C CD28–/KLRG1+	*n* = 16 **younger** males, 20–34 years. *n* = 16 **older** males, 50–64 years. **Included:** non-smokers, no drugs, infection free 6 weeks prior.	• Blood samples: at rest, immediately, and 1 h post exercise. • VO_2_max • Height, Body mass	Acute Exercise—cycling. 30 min cycling at 80–85% VO_2_max. Submaximal cycling test to determine VO_2_max.	1. Exercise mobilizes Ts. 7-fold ↑ Ts cells immediately after; levels returned to pre-exercise values 1 h post.
Spielmann et al., 2011 C CD28–/KLRG1+ (Ts_1_) and CD57+/KLRG1+ (Ts_2_)	*n* = 102 males, 18–61 years. **Included:** healthy, non-smoker. **Excluded:** excessive alcohol, immune/bp drugs, routinely using ibuprofen/aspirin, anti-depressants, hormone replacement, chronic/acute infection.	• Resting blood sample • VO_2_max • Body fat, PA level questionnaire	Acute Exercise—cycling. Submaximal incremental cycling exercise to determine VO_2_max.	1. VO_2_max and Ts_1_ association withstood age adjustment. 2. VO_2_max and Ts_2_ inversely associated. Age and Ts_2_ no longer associated when VO_2_max adjusted. 3. Above avg. VO_2_max, Ts_2_ 37% < avg. VO_2_max. Avg. ≈ below avg.
Simpson et al., 2010 I/C CD28–/KLRG1+ (Ts_1_) and CD57+/KLRG1+ (Ts_2_)	*n* = 9 males, 20–35 years. **Included:** healthy, non-smokers, free from illness 6 weeks prior to testing.	• Blood samples: at rest, immediately after, and 1 h post exercise. • VO_2_max	Acute Exercise—running. Treadmill-running protocol at 80% VO_2_max until exhaustion. Incremental treadmill-running protocol to determine VO_2_max.	1. Both Ts ↑ immediately after exercise; Ts_2_ ↑ 1.9-fold. 2. Both Ts ↓ 1 h post exercise, approaching pre-exercise levels.
Cosgrove et al., 2012 I CD57+/KLRG1+	Triathletes training for ironman competition. *n* = 10 (9 males, 1 female), 39–50 years.	• 6x blood samples over 6-months: baseline, 4x training, and post-race. • Max O_2_ uptake • HR	Repeated exercise—triathlon training.	1. No change in Ts over time.
Theall et al., 2020 I CD57+/KLRG1+	*n* = 15 females, 20 years (V1, V3). *n* = 9 females, 20 years (V2). **Included:** healthy swimmers.	• Blood samples: at rest and immediately post exercise, at V1–3. • HR, blood lactate, self-report questionnaires	Repeated and Acute Exercise—swimming. Incrementally loaded 22.86 m swims until withdrawal or failure to make forward progress. Conducted at: • V1 = after championships—mod training • V2 = off-season—mod training • V3 = start of new season—high training	1. Trend for Ts mobilization with Ts %↑ post exercise. 2. No change in Ts % from V1–3.

*The study reference, listed in the first column, is the first author and date published. The study design was defined as either: cross-sectional (C), intervention (I), randomized controlled trial (RCT), or intervention and cross-sectional (I/C). The CD8^+^ Ts cell populations are outlined in the first column. The study cohorts are briefly described under the column “Cohort.” The column “Data collection” outlines the methods used to collect data. If an exercise intervention was performed in the study, it is described in the “Exercise intervention” column as either acute or repeated exercise with the type (predominantly cycling or running). The “Outcome” column lists the studies' findings relating to the influence of exercise on the defined Ts cell population, where # = number/count and % = proportion/percentage*.

**Table 3:** Summary of results concerning the influence of exercise on exhausted T (Tex) cells from included studies in this systematic review.

**Reference** **Study design** **CD8+ Tex population**	**Cohort**	**Data collection**	**Exercise intervention**	**Outcome**
Dorneles et al., 2020 I PD-1+	*n* = 8 sedentary normoglycemic obese males, 25–30 years. **Excluded:** immune, cardiac, endocrine, or metabolic disease/drugs/supplements.	• Blood samples: pre and post exercise. • VO_2_max • maxVelocity	Acute Exercise—running. High intensity interval exercise (10 × 60 s at 85–90% maxVelocity alternated with 75 s recovery at 50 s maxVelocity on treadmill) and exhaustive exercise (stepping up/down), a week apart.	1. Exercise induced Tex mobilization. 2. In post-exercise serum, Tex ~43% > pre-exercise serum; regardless of intervention.
Schenk et al., 2021 RCOT PD-1+	*n* = 24 males, 20–35 years. • RE ± EE: *n* = 13. • EE ± RE: *n* = 13. ** Excluded:** contraindications to physical exercise, drug intake in the last 6 weeks.	• Blood samples: at rest, immediately after, and 1 h post. • VO_2_peak, 1RM • FACS, MFI	Acute Exercise—cycling. Endurance exercise (EE) = 45 min at 60% VO_2_ peak, with 5 min warm-up. Resistance exercise (RE) = 4 × sets 8–10 reps at 70% 1RM at each machine. Incremental cycling until exhaustion to determine VO_2_ peak.	1. Tex mobilization in EE > RE.
Wadley et al., 2020 I PD-1+	*n* = 8 males, 29 ±5 years. **Excluded:** smokers, use of antioxidant vitamin supplements or anti-inflammatory drugs in last 8 weeks, viral/bacterial infections in last 4 weeks.	• Blood samples: at rest, immediately after, 0.5 h post and 1 h post. • VO_2_max • HR, BM	Acute Exercise—cycling. Participants underwent two interventions a week apart: 1. MOD = cycling at 60% VO_2_max for 58 min. 2. HIIE = 10 × 4 min intervals at 85% VO_2_max, 2 min rest intervals. Cycling ramp test to exhaustion to determine VO_2_max.	1. Tex mobilized in both MOD and HIIE.
van der Geest et al., 2017 I PD-1+ (Tex_1_) and CTLA-4+ (Tex_2_)	*n* = 20 males and females, 80 years. Recruited from mass participation walking event.	• Blood samples: pre and post (10 min) event. • HRmax • BMI • Lean body mass	Repeated Exercise—walking. Walk 30 km per day (24–27°C) for 4 consecutive days, 70% intensity = mod intensity.	1. Tex_1_ unchanged. 2. After exercise, ↑% Tex_2_ in effector memory and terminally differentiated T cells.
Gustafson et al., 2017 I/C PD-1+ (Tex_1_) and CTLA-4+ (Tex_2_)	*n* = 15 males, 31 ± 4 years. • Active, *n* = 10 • Sedentary, *n* = 5 ** Included:** healthy, non-smokers, no known cardiopulmonary or immune disease, not taking steroids or immune modulating drugs.	• Blood samples: at rest, immediately after, 3 h post and 24 h post exercise. • VO_2_max • Tracked activity level	Acute Exercise—cycling. Cohort completed both: • Endurance cycling protocol-−45 min at 60% max-workload. • Incremental maximal cycling test.	1. Intervention mobilized Tex_1_. 2. Sedentary Tex_1_ > active Tex_1_. 3. No change in Tex_2_ from intervention.
Cury-Boaventura et al., 2018 I CTLA4+	*n* = 16 males, 26 years. **Included:** professional futsal athletes for at least 10 years, 4 h futsal training × 5/week. **Excluded:** history of infection, viruses, chronic lesions, diabetes, rheumatoid arthritis, hormonal dysfunction, lupus, or other inflammatory or hematological diseases, taking medication.	• Blood samples: pre and post exercise.	Acute Exercise—futsal game 2 × (5 min futsal + 5 min recovery).	1. After exercise, no change in Tex.
Wong et al., 2021 RCT CTLA-4+	Community-dwelling males and females, 60–85 years. Horticultural therapy (HT), *n* = 22 • Control group (CON), *n* = 24 **Excluded:** severe psychiatric disorders, medical history of stroke, epilepsy, ischemic heart disease, heart failure, chronic obstructive pulmonary disease, cancer, liver failure, and thyroid disorder, upper and lower limb motor difficulties, and significant visual or hearing impairment, undergoing any concurrent therapy, including consumption of medication(s).	• Blood samples: pre, 3^rd^, and 6^th^ month post.	Repeated Exercise—walking. 2-arm, single blind HT = 15 h sessions over 6 months. Sessions 1–12 were weekly, and 13–15 were monthly. • CON = waitlist to conduct the HT program post RCT.	1. HT ↓ Tex in TEMRA (CD45RA+/CD27–) cells.

*The study reference, listed in the first column, is the first author and date published. The study design was defined as either: cross-sectional (C), intervention (I), randomized controlled trial (RCT), randomized cross-over trial (RCOT), or intervention and cross-sectional (I/C). The CD8^+^ Tex cell populations are outlined in the first column. The study cohorts are briefly described under the column “Cohort.” The column “Data collection” outlines the methods used to collect data. If an exercise intervention was performed in the study, it is described in the “Exercise intervention” column as either acute or repeated exercise with the type (predominantly cycling or running). The “Outcome” column lists the study's outcomes relating to the influence of exercise on the defined Tex cell population, where # = number/count and % = proportion/percentage*.

### Quality Assessment

National Institutes of Health (NIH) quality assessment (QA) tools were used to assess the quality of the included studies. The majority of studies were deemed high quality (*n* = 21), nine studies were rated moderate quality, and no studies were considered low quality. Common limitations included small sample size, absence of participant blinding, and lack of multiple outcome/exposure measurements. Among the moderate quality studies, additional limitations included only modest descriptions of interventions/exposures, exposure not measured prior to outcome, single level/amount of exposure examined, short timeframe for association between intervention/exposure and outcome, and inadequate statistical analysis, particularly in adjustment(s) for confounding variables. Supplementary Table 4 contains detailed scores for each included study.

### Senescent CD8^+^ T Cells

#### Acute Exercise Induces Mobilization of Senescent CD8^+^ T Cells

Most studies investigating senescent CD8^+^ T cells after acute exercise reported that acute exercise mobilized senescent T cells into the periphery (*n* = 16). This common conclusion was demonstrated by a transient increase in senescent T cell numbers immediately after exercise, followed by numbers returning to pre-exercise levels approximately 1–3 h after exercise (Simpson et al., 2010; Turner et al., 2010; Wang et al., 2011; Bigley et al., 2012; Brown et al., 2014; Lavoy et al., 2014; LaVoy et al., 2017; Spielmann et al., 2014; Ingram et al., 2015; Krüger et al., 2016; Cury-Boaventura et al., 2018; Karim and Jabbar, 2018; Minuzzi et al., 2018; Ross et al., 2018; Curran et al., 2019; Theall et al., 2020). Two exceptions to this finding were in acute exercise studies that only measured senescent T cells at rest, and thus could not report mobilization (Spielmann et al., 2011; Bastos et al., 2020). Among the studies demonstrating mobilization, a variety of protein surface markers were used to define the senescent T cells including: KLRG1^+^ (*n* = 5); CD28^−^ (*n* = 3); CD28^−^/CD57^+^ (*n* = 2); CD28^−^/CD27^−^ (*n* = 3); CD28-/KLRG1^+^ (*n* = 3); and CD57^+^/KLRG1^+^ (*n* = 2). Aerobic exercise in the form of cycling (*n* = 10), running (*n* = 4), swimming (*n* = 1), and futsal (*n* = 1) were employed as the type of acute exercise. Cohort characteristics ranged from male to female, athletes to sedentary, and young to older adults. Overall, regardless of the protein surface markers used to define senescent T cell populations, and cohort characteristics such as sex, physical activity level, and age, acute aerobic exercise generally mobilized senescent CD8^+^ T cells.

One of the studies demonstrated that in an older cohort only, CD28^−^ senescent T cells were preferentially mobilized in response to exercise compared to CD28^+^ T cells (Ross et al., 2018). Although a comparable study with a similar aged cohort, does not report this finding, their results (in the first figure under CMV seronegative individuals for direct comparison) illustrate a similar trend (Simpson et al., 2011). Both studies indicate that age does not impact the ingress and subsequent egress induced by acute exercise. However, CMV serostatus can influence exercise induced mobilization of senescent T cell such that CMV seropositive individuals have a greater ingress and egress of senescent T cells after acute exercise (Turner et al., 2010; Simpson et al., 2011; Bigley et al., 2012; Lavoy et al., 2014; LaVoy et al., 2017). Exercise intensity can also alter mobilization whereby greater exercise intensity resulted in greater mobilization of senescent T cell, and other T cell populations (Krüger et al., 2016; LaVoy et al., 2017). Therefore, under certain conditions such as advanced age, CMV seropositivity and high exercise intensity, senescent CD8^+^ T cells may be preferentially mobilized in response to acute exercise.

#### Increased Cardiorespiratory Fitness Can Protect Against Senescent CD8^+^ T Cells

Increased cardiorespiratory fitness can protect against the production/accumulation of senescent CD8^+^ T cells populations. Individuals with an above-average cardiorespiratory fitness measured by VO_2_max (47.3 ml/kg/min) had 37% fewer KLRG1^+^/CD57^+^ and KLRG1^+^/CD28^−^ senescent T cells than individuals with an average or below-average VO_2_max (43.0 and 34.4 ml/kg/min, respectively) (Spielmann et al., 2011). A cross-sectional study demonstrated a similar result where master athletes had accumulated less KLRG1^+^ senescent T cells than aged-matched sedentary controls (Minuzzi et al., 2018). The VO_2_max of the master athletes (40.4 ml/kg/min), aged between 40 and 60 years old, was less than the above-average VO_2_max in the previous study, which included participants aged from 18 to 61 years; this variation likely relates to the known decline of VO_2_max with age (Rogers et al., 1990; Bradshaw et al., 2005; Spielmann et al., 2011; Minuzzi et al., 2018). However, the Spielmann at al. study demonstrated that the relationship between VO_2_max and senescent T cells withstood adjustment for age (Spielmann et al., 2011). In summary, several studies demonstrate a trend toward increased cardiorespiratory fitness protecting against the accumulation of senescent CD8^+^ T cells; however, more research is required to confirm this finding.

#### Endurance Resistance Training Can Reduce Senescent CD8^+^ T Cells in Sedentary Older Adults

Resistance training exercise interventions were shown to decrease senescent CD8^+^ T cells populations. Resistance training over a 6- and 12-week intervention reduced the proportion of CD28^−^/CD57^+^ and number of CD28^−^ senescent T cells, respectively (Shimizu et al., 2011; Cao Dinh et al., 2019). The cohorts of both studies consisted of sedentary older adults aged 61–79 years. The 6-week intervention only altered CD28^−^/CD57^+^ senescent T cells in individuals performing strength endurance training, and not intensive strength training. The strength endurance training more closely resembled the 12-week intervention in the Shimizu et al. study (Shimizu et al., 2011; Cao Dinh et al., 2019). Therefore, repeated endurance resistance training appears to reduce senescent CD8^+^ T cells in sedentary older adults >60 years.

#### Cohort Characteristics Associated With Unchanged Senescent CD8^+^ T Cells

It is well-established that senescent T cells accumulate with age (Crespo et al., 2013), not surprisingly, studies with a young (under 30 years old) cohort typically do not report any differences in senescent T cell populations with exercise, as the senescent T cell populations are likely too small for meaningful changes to be detected (Wang et al., 2011; Karim and Jabbar, 2018; Theall et al., 2020). However, the two Brown et al. (2014, 2015) studies both contained a young cohort yet only one demonstrated a change in senescent T cells between trained and untrained individuals (Brown et al., 2014, 2015). The contrasting result is likely due to differences in physical activity level of the “untrained” individuals. The study which showed no change in senescent T cells included “untrained” individuals who were physically active for 3.6 hours per week, compared to 2.0 hours per week (results for females only for direct comparison) in the other study (Brown et al., 2014, 2015). Other studies consisting of physically fit cohorts demonstrated similar results where senescent T cells were unchanged after exercise (Cosgrove et al., 2012; Silva et al., 2016; Bastos et al., 2020). Overall, it appears that acute and/or repeated exercise resulting in no change to senescent CD8^+^ T cells is largely related to cohort characteristics such as young age and high physical fitness.

#### Long-Term Exercise May Maintain Senescent CD8^+^ T Cells Telomere Length

Senescent CD8^+^ T cells are known to have shorter telomeres, with cross-sectional data indicating that CD28^+^ cells have longer telomeres than CD28^−^ T cells (Silva et al., 2016). Participants who had not engaged in training in the previous 5 years had ~15–20% shorter CD8^+^ and senescent T cell telomeres than those who had participated regularly in exercise and physical activity. Although the number of senescent CD8^+^ T cells was unchanged in this study, it appears that exercise may protect against senescence through the maintenance of telomere length (Silva et al., 2016).

#### The Influence of Repeated Exercise on Senescent CD8^+^ T Cells Impacts Cytokine Levels

The impact of repeated exercise on senescent CD8^+^ T cells appeared to extend beyond reducing cell numbers, and into restoring immune function mediated by cytokines (Wang et al., 2011). Interferon-γ (IFN-γ) levels increased, and interleukin-6 (IL-6) and plasma myeloperoxidase (MPO) levels decreased when senescent T cell numbers decreased. Furthermore, when senescent T cell numbers remained stable, these cytokine levels were also stable (Wang et al., 2011; Silva et al., 2016; Duggal et al., 2018). Therefore, the ability of repeated exercise to limit senescent CD8^+^ T cell accumulation appears to have functional consequences through alterations in blood cytokine concentrations.

### Exhausted CD8^+^ T Cells

#### Acute Exercise Induces Mobilization of PD-1^+^ and May Not in CTLA-4^+^ CD8^+^ T Cells

Similar to senescent CD8^+^ T cell populations, acute exercise induced the mobilization of PD-1^+^ exhausted CD8^+^ T cells into the peripheral blood compartment (Gustafson et al., 2017; Dorneles et al., 2020; Wadley et al., 2020; Schenk et al., 2021). Furthermore, blood serum collected after acute exercise induced CD8^+^ T cells to express exhaustion marker PD-1 at a greater rate (~43%) than pre-exercise blood serum (Dorneles et al., 2020). Mobilization of exhausted T cells was not reported in repeated exercise studies due to their study design (van der Geest et al., 2017; Wong et al., 2021). Acute exercise studies investigating CTLA-4^+^ exhausted T cells demonstrated unchanged levels in response to exercise, and thus no mobilization (Gustafson et al., 2017; Cury-Boaventura et al., 2018). Unlike senescent CD8^+^ T cells, the mobilization of exhausted CD8^+^ T cells may be dependent on the surface protein markers.

Similar to senescent CD8^+^ T cells, the type and intensity of acute exercise influenced the mobilization of PD-1^+^ exhausted T cells. High intensity and exhaustive exercise mobilized PD-1^+^ T cells greater than moderate intensity and endurance exercise, respectively (Gustafson et al., 2017; Wadley et al., 2020). Furthermore, endurance exercise mobilized PD-1^+^ T cells to a greater extent than resistance exercise (Schenk et al., 2021).

#### Sedentary Lifestyle May Increase Exhausted CD8^+^ T Cells

Three studies investigated the effects of repeated exercise on PD-1^+^ and CTLA-4^+^ exhausted CD8^+^ T cells; two indicated that a sedentary lifestyle is linked to an increase in exhausted T cells. Cross-sectional data demonstrated that sedentary adult males, participating in <1 hour of scheduled physical activity per day, had more PD-1^+^ exhausted T cells than active individuals (Gustafson et al., 2017). After a 6-month horticultural therapy which included walking as an exercise intervention, older adults aged 58–76 years had less CTLA-4^+^ exhausted T cells than a control group not participating (Wong et al., 2021). Physical activity levels were not measured in the intervention study, thus it cannot be determined if the control group were truly sedentary (Wong et al., 2021). The third study investigating repeated exercise reported an increase in CTLA-4^+^ exhausted T cells after a 4-day walking event (van der Geest et al., 2017). The older adult participants were physically active (mean physical activity hours/week: males 8.5 ± 8.0, females 5.8 ± 5.9) and likely trained to walk the 30 km each day at the event. Overall, a sedentary lifestyle may lead to an increase in exhausted CD8^+^ T cells.

## Discussion

A systematic search of the literature identified 30 studies that investigated the impact of exercise on protein-surface-marker-defined senescent and/or exhausted CD8^+^ T cell populations. Only 30% (*n* = 7) of eligible studies examined exhausted CD8^+^ T cells, highlighting the lack of relevant studies on this cell type. Overall, it appears that acute exercise can induce mobilization of senescent and exhausted CD8^+^ T cells. The influence of repeated exercise on limiting senescent/exhausted CD8^+^ T cells includes: increased cardiorespiratory fitness may protect against the accumulation of senescent CD8^+^ T cells, endurance resistance training appears to reduce senescent CD8^+^ T cells in sedentary older adults, and a sedentary lifestyle may lead to an increase in exhausted CD8^+^ T cells. However, the number of studies available to conceive these findings was limited such that only two studies were designed appropriately to possibly generate the results in each of the three previous findings. More research is required to confirm the influence of repeated exercise on senescent and exhausted CD8^+^ T cells.

A limited number of studies have investigated the influence of repeated exercise on senescent and exhausted T cells. The shortage of evidence-based investigations on this topic can in part be attributed to the wide variety of terminologies, and inconsistent definitions, for senescent and exhausted T cells. The requirement for senescent and exhausted CD8^+^ T cell populations to be defined by protein surface markers restricted the inclusion of some studies in this review. The terminology and definitions of exhausted and senescent T cell populations must be clarified and employed systematically in future studies. Additional investigation will be required to determine the clinical relevance of the underlying relationships between repeated exercise and senescent and exhausted T cells. A clearer understanding of these relationships will inform the development of more effective treatment and management strategies for healthy aging, cancer prevention, and vaccine efficiency.

Exercise can mobilize senescent and exhausted CD8^+^ T cells immediately after an acute bout of physical activity, with levels returning to normal shortly after. It is well-established that exercise elicits transient leukocytosis; however, senescent and exhausted T cells may be preferentially mobilized under certain conditions (Deuster et al., 1988; Turner and Brum, 2017; Ross et al., 2018). Both senescent and exhausted T cells were shown to increase in mobilization with higher intensity exercise (Krüger et al., 2016; Gustafson et al., 2017; LaVoy et al., 2017; Wadley et al., 2020; Schenk et al., 2021). Additionally, older or CMV seropositive individuals demonstrated preferential mobilization of senescent T cells only, likely due to a lack of studies investigating exhausted T cells (Turner et al., 2010; Simpson et al., 2011; Bigley et al., 2012; Lavoy et al., 2014; LaVoy et al., 2017; Ross et al., 2018). Other studies, with differing terminology, demonstrate similar findings such that terminally differentiated lymphocytes exhibiting a senescent phenotype can be selectively mobilized after an acute bout of exercise (Cao Dinh et al., 2016). Unlike PD-1^+^ exhausted CD8^+^ T cells, CTLA-4^+^ exhausted CD8^+^ T cells were unchanged after acute exercise. A study exposing CD8^+^ T cells to exercise-induced metabolites supports this outcome (Rundqvist et al., 2020). Further research is required to understand, and confirm, the mobilization of differing exhausted CD8^+^ T cells in response to acute exercise.

Lymphocytes mobilized by acute exercise are more sensitive to apoptosis through intrinsic and extrinsic apoptotic pathways explained in detail in a review by Simpson (2011). The removal of lymphocytes, and specifically senescent T cells, purportedly creates “immune space” for the production of naïve T cells which are more immunologically responsive. It is yet to be confirmed whether senescent T cells undergo apoptosis at a greater rate than other T cell populations. Senescent T cells can be resistant to some apoptotic pathways, but evidence indicates that exercise-induced apoptotic pathways involve oxidative stress where senescent T cells may be less tolerant than other T cell populations. Even so, if senescent T cells are preferentially mobilized it is likely they are also exposed to apoptotic pathways. Frequent bouts of acute exercise and subsequent T cell shifts may have an accumulative long-term restorative effect on the immune system by “making space” for naïve T cells to increase the TCR repertoire (Simpson, 2011). Therefore, the mobilization of senescent and (some) exhausted T cells may account for the benefits of repeated exercise.

A secondary outcome of this systematic review was that senescent CD8^+^ T cells may be influenced by sex. Males appear to have a higher proportion of CD28^−^/CD57^+^ senescent CD8^+^ T cells than females (Brown et al., 2014). The influence of sex on the immune response and cells is well-established; however, studies investigating the regulation of senescent T cells are lacking, and outcomes of current studies are inconsistent (Al-Attar et al., 2016). Some studies report that males have greater numbers of senescent T cells than females (Hirokawa et al., 2013; Di Benedetto et al., 2015; Nacka-Aleksić et al., 2020), whereas other studies did not identify substantial differences between men and women (Al-Attar et al., 2016; Reed et al., 2019). One of the studies showing a higher number of senescent T cells in males proposed that the sex hormone estrogen may regulate longevity, thus protecting females from age-related immune decline, such as the loss of surface marker CD28 (Hirokawa et al., 2013). Future studies investigating senescent T cell populations should account for sex as a potential confounder, and employ the appropriate experimental or statistical controls.

It is recognized that mechanisms other than apoptosis may be behind the beneficial effect of repeated exercise limiting senescent and exhausted CD8^+^ T cell accumulation. Other possible mechanisms are detailed elsewhere and may include exercise-induced delayed thymic atrophy (Duggal et al., 2018) or engagement in repeated exercise reflecting a generally healthier lifestyle including better nutrition (Ravaglia et al., 2000; Wu et al., 2019). Another limitation in the review is the assumption that cardiorespiratory fitness is the sole result of repeated exercise. Cardiorespiratory fitness is a heritable trait and genetic factors determined ~70% of the relationship between exercise and an individual's VO_2_max score (Mustelin et al., 2011). Future studies will need to determine that those with increased cardiorespiratory fitness and low senescent CD8^+^ T cells numbers is related to exercise, and not due to genetic factors predisposing the person to delayed immunosenescence, and a high VO_2_max score. Future work should explore whether a sedentary lifestyle leads to increased accumulation of senescent CD8^+^ T cells, and evaluate exercise and physical activity interventions that could protect against immunosenescence.

## Future Directions

Approaches to reversing exhausted and senescent CD8^+^ T cell phenotype are emerging, however prevention remains important as accumulation of these cells is linked to decreased vaccine efficiency and increased cancer risk linked to impaired immunosurveillance (Bigley et al., 2013; Pawelec, 2019; Thomas et al., 2020). Exercise has been investigated as an adjunct therapy for improving both vaccination and cancer outcomes (Simpson et al., 2012; Neilson et al., 2014; Campbell and Turner, 2018; Duggal et al., 2019; Hwang et al., 2020). A meta-analysis reported that an active lifestyle involving high levels of leisure activities can reduce all-cause cancer rates by 46% (Bigley et al., 2013). Single bouts as well as habitual exercise before vaccination can improve immune responses, and thus vaccine efficiency (Duggal et al., 2019). However, few studies have examined this relationship with a focus on senescent and/or exhausted CD8^+^ T cells. The lack of evidence makes it difficult to establish the mechanistic or clinical relevance of regular physical exercise programs for clinical applications and the general population. One possible mechanism details the ability of intense cardiorespiratory exercise to induce an epigenetic mechanism, microRNA expression, to regulate telomere homeostasis in white blood cells to improve immune function and physical health (Chilton et al., 2014). This mechanism would benefit senescent T cells given their known shortened telomeres, as individuals engaging in exercise have longer telomeres (Silva et al., 2016). However, the mechanism was demonstrated in a heterogenous white blood cell population, and future studies will need to isolate senescent CD8^+^ T cells. This mechanism could be responsible for the ability of exercise to maintain telomere lengths, and consequently improve immune function. As well as identifying the mechanism(s) underlying the benefits of exercise, future studies should determine whether this impact is clinically meaningful, particularly in senescent-prone individuals such as the older adults or cancer patients.

Future work should investigate if epigenetics can provide an insight into the association between latent CMV infection and increased numbers of senescent CD8^+^ T cells (Simpson et al., 2012). Several of the included studies in this review have detailed the influence of CMV on senescent CD8^+^ T cells. The chronic stimulation of cytotoxic T cells from CMV infection drives their senescence, and some researchers describe the same model for CMV's association with exhausted cytotoxic T cells (Simpson et al., 2016). We propose an epigenetic mechanism, specifically DNA methylation, that regulates this model. Upon responding to a viral infection, cytotoxic T cells undergo genome-wide DNA methylation remodeling toward an effector phenotype (Schlums et al., 2015). The DNA methylome of CD28^−^ senescent T cells are unique to other T cell phenotypes and contributes to their functional characteristics (Suarez-Álvarez et al., 2016). Therefore, we consider that the chronic stimulation of CMV drives DNA methylation remodeling within cytotoxic T cells, and results in the generation of senescent T cells. A similar theory has been proposed for natural killer (NK) cells, but data are sparse for CD8^+^ T cells (Schlums et al., 2015). Understanding the molecular mechanism underlying senescent T cell development driven by CMV infection may lead to novel interventions to prevent senescent T cell accumulation. Most importantly, future studies should account for the association between CMV and senescent T cells.

The immunological continuum throughout life needs to be fully characterized to develop personalized and predictive medicine approaches to optimize immune health. The immune risk profile describes a set of immunological parameters that currently predict morbidity and mortality; however, the profile is restricted to immune cell ratios and limited protein biomarkers (Simpson and Guy, 2010). Cutting edge single-cell technologies should provide in-depth profiling of immune cell populations that generate a signature of immune decline over time. Algorithmic frameworks that combine multiparametric lifestyle data and molecular markers are required to develop personalized immune medicine approaches. These frameworks will consist of tests that quantify immune decline. Personalized medicine is already a reality in some areas of healthcare, such as cancer therapy, where lifestyle or pharmacology interventions are tailored to the individual. In the case of declining immune health, these interventions could boost vaccine efficiency or prevent cancer. Personalized and predictive medicine should be accompanied by new clinical/practical guidelines on managing immune health and immunosenescence.

## Conclusion

Studies show that repeated exercise can be beneficial in limiting the number of senescent and exhausted CD8^+^ T cells. Exercise-induced preferential mobilization of senescent CD8^+^ T cells, and the subsequent promotion of apoptosis in the peripheral blood compartment, is the proposed underlying mechanism. The clinical relevance of the effect of exercise on exhausted and senescent CD8^+^ T cells has yet to be determined, but with effective clinical and lifestyle implementation could prevent cancer and improve vaccine efficiency. Future studies investigating the effects of exercise should focus on clearly defining the target exhausted and senescent CD8^+^ T cell populations, and control for confounding variables such as sex and CMV status.

## Data Availability Statement

The original contributions presented in the study are included in the article/Supplementary Material, further inquiries can be directed to the corresponding author/s.

## Author Contributions

TD conducted the systematic search and drafted the manuscript. TD and DP screened articles. DP, SR, WT, and AB revised and edited the manuscript. All authors contributed to the article and approved the submitted version.

## Conflict of Interest

The authors declare that the research was conducted in the absence of any commercial or financial relationships that could be construed as a potential conflict of interest.

## Publisher's Note

All claims expressed in this article are solely those of the authors and do not necessarily represent those of their affiliated organizations, or those of the publisher, the editors and the reviewers. Any product that may be evaluated in this article, or claim that may be made by its manufacturer, is not guaranteed or endorsed by the publisher.

## Abbreviations

CMV: cytomegalovirus
IRP: immune risk profile
IFN-γ: interferon-γ
IL-6: interleukin-6
NIH: National Institutes of Health
MPO: plasma myeloperoxidase
NK: natural killer
PRISMA: preferred reporting items for systematic reviews and meta-analyses
QA: quality assessment
Tex: exhausted T cell
Ts: senescent T cell
VO_2_max: maximal aerobic capacity.
