# Supplementary material for: Influence of Exercise on Exhausted and Senescent T Cells: A Systematic Review
Source: Front Physiol. 2021 Aug 20;12:668327. doi: 10.3389/fphys.2021.668327 (PMC8417807; doi:10.3389/fphys.2021.668327)
Supplement: Supplementary file 1 [file Data_Sheet_1.PDF]

Supplementary Material

Supplementary Table S1: Systematic review search strategy for each data base.

Database	PubMed	Embase	Web of Science
**Search string**	("t cell*"[All Fields] OR "t lymphocyte*"[All Fields])<br><br>AND ("KLRG1"[All Fields] OR "CD28"[All Fields] OR "CD57"[All Fields] OR "CTLA"[All Fields] OR "PD1"[All Fields] OR "TIM3"[All Fields] OR "LAG3"[All Fields] OR "senesc*"[All Fields] OR "exhaust*"[All Fields])<br><br>AND ("Sports"[MeSH Terms] OR "physical activit*"[All Fields] OR "exercis*"[All Fields])<br><br>AND (2010:2021[pdat])	('t cell*' OR 't lymphocyte*')<br><br>AND ('immunosenescence'/exp OR 'senesc*' OR 'exhaust*' OR 'klrg1' OR 'cd28' OR 'cd57' OR 'ctla' OR 'pd1' OR 'tim3' OR 'lag3')<br><br>AND ('physical activity'/exp OR 'sport'/exp OR 'exercis*' OR 'physical activit*')<br><br>AND [embase]/lim<br><br>AND [2010-2021]/py	(TS="T cell*" OR TS="T lymphocyte*")<br><br>AND (TS="KLRG1" OR TS="CD28" OR TS="CD57" OR TS="CTLA" OR TS="PD1" OR TS="TIM3" OR TS="LAG3" OR TS="senesc*" OR TS="exhaust*")<br><br>AND (TS="physical activit*" OR TS="exercis*" OR TS="sport*")<br><br>AND (PY=2010-2021)

Supplementary Table S2: The National Institutes of Health (NIH) quality assessment tool for observational cohort and cross-sectional studies and the NIH quality assessment tool for before-after (pre-post) studies with no control group were adapted to create a single quality assessment tool. The adapted tool was used to assess the quality of intervention and/or cross-sectional studies. The tool's criteria are described with possible responses listed. Each criterion is numbered and corresponds to a specific study type (i.e., intervention and/or cross-sectional).

Index	Study Type	Criteria<br>Response options: Yes/ No/ Cannot Determine/ Not Applicable/ Not Reported
1	Both	Was the research question or objective clearly stated?
2	Both	Were eligibility/selection criteria for the study population prespecified and clearly described?
3	Intervention	a) Were the participants in the study representative of those who would be eligible for the test/service/intervention in the general or clinical population of interest? Were all eligible participants that met the prespecified entry criteria enrolled?
	Cross-sectional	b) Were all the subjects selected or recruited from the same or similar populations (including the same time period)? Were inclusion and exclusion criteria for being in the study prespecified and applied uniformly to all participants?
4	Both	Was the sample size sufficiently large to provide confidence in the findings? Was a sample size justification, power description, or variance and effect estimates provided?
5	Intervention	a) Was the test/service/intervention clearly described and delivered consistently across the study population?
	Cross-sectional	b) Were the exposure measures (independent variables) clearly defined, valid, reliable, and implemented consistently across all study participants?
6	Cross-sectional	For the analyses in this paper, were the exposure(s) of interest measured prior to the outcome(s) being measured?
7	Cross-sectional	For exposures that can vary in amount or level, did the study examine different levels of the exposure as related to the outcome (e.g., categories of exposure, or exposure measured as continuous variable)?
8	Both	Was the timeframe sufficient so that one could reasonably expect to see an association between exposure/intervention and outcome if it existed?
9	Both	Were the outcome measures prespecified, clearly defined, valid, reliable, and assessed consistently across all study participants?
10	Both	Were the people assessing the outcomes blinded to the participants' exposures/ interventions?
11	Both	Was the loss to follow-up after baseline 20% or less? Were those lost to follow-up accounted for in the analysis?
12	Intervention	a) Were outcome measures of interest taken multiple times before the intervention and multiple times after the intervention (i.e., did they use an interrupted time-series design)?
	Cross-sectional	b) Was the exposure(s) assessed more than once over time?
13	Intervention	Did the statistical methods examine changes in outcome measures from before to after the intervention? Were statistical tests done that provided p values for the pre-to-post changes?
14	Intervention	a) If the intervention was conducted at a group level (e.g., a whole hospital, a community, etc.), did the statistical analysis take into account the use of individual-level data to determine effects at the group level?
	Cross-sectional	b) Were key potential confounding variables measured and adjusted statistically for their impact on the relationship between exposure(s) and outcome(s)?
**Quality Rating –** Good, Fair or Poor		

Supplementary Table S3: The National Institutes of Health (NIH) quality assessment tool of controlled intervention studies was used to assess the quality of included randomized controlled trial (RCT) and a randomized cross-over trial (RCOT). The tool's criteria are described and numbered with possible responses listed.

**Index**	**Criteria**<br>Response options: Yes/ No/ Cannot Determine/ Not Applicable/ Not Reported
1	Was the study described as randomized, a randomized trial, a randomized clinical trial, or an RCT?
2	Was the method of randomization adequate (i.e., use of randomly generated assignment)?
3	Was the treatment allocation concealed (so that assignments could not be predicted)?
4	Were study participants and providers blinded to treatment group assignment?
5	Were the people assessing the outcomes blinded to the participants' group assignments?
6	Were the groups similar at baseline on important characteristics that could affect outcomes (e.g., demographics, risk factors, co-morbid conditions)?
7	Was the overall drop-out rate from the study at endpoint 20% or lower of the number allocated to treatment?
8	Was the differential drop-out rate (between treatment groups) at endpoint 15 percentage points or lower?
9	Was there high adherence to the intervention protocols for each treatment group?
10	Were other interventions avoided or similar in the groups (e.g., similar background treatments)?
11	Were outcomes assessed using valid and reliable measures, implemented consistently across all study participants?
12	Did the authors report that the sample size was sufficiently large to be able to detect a difference in the main outcome between groups with at least 80% power?
13	Were outcomes reported or subgroups analyzed prespecified (i.e., identified before analyses were conducted)?
14	Were all randomized participants analyzed in the group to which they were originally assigned, i.e., did they use an intention-to-treat analysis?
**Quality Rating –** Good, Fair or Poor	

Supplementary Table S4: Quality of the included studies was assessed using an adapted NIH quality assessment tool (described in Supplementary Table S2), the responses to each criterion and the overall quality rating. Included studies are listed under 'reference' with the first author and year of publication provided. The 'study design' describes whether the study was an intervention (I), cross-sectional analysis (C), both an intervention and cross-sectional analysis (I/C), a randomized controlled trial (RCT), or a randomized cross-over trial (RCOT). The tool's criteria are listed by index number, descriptions are detailed in Supplementary Table S2 and S3.

**Studies**		**QA tool criteria**														**Quality Rating**
**Reference**	**Study design**	**1**	**2**	**3**	**4**	**5**	**6**	**7**	**8**	**9**	**10**	**11**	**12**	**13**	**14**	
Bastos 2020	C	Yes	Yes	Yes	No	No	No	Yes	No	Yes	No	Cannot Determine	Yes	n/a	Yes	Fair
Bigley 2012	I	Yes	Yes	Yes	No	Yes	n/a	n/a	Yes	Yes	No	Cannot determine	No	Yes	Nos	Good
Brown 2014	I/C	Yes	Yes	Yes	No	Yes	Yes	Yes	Yes	Yes	No	Cannot determine	No	Yes	Yes	Good
Brown 2015	I/C	Yes	Yes	Yes	No	Yes	No	Yes	No	Yes	No	Cannot Determine	No	Yes	Yes	Fair
CaoDinh 2019	RCT	Yes	Yes	Yes	No	Yes	Yes	Yes	Yes	Yes	Yes	Yes	Cannot Determine	Yes	Yes	Good
Curran 2019	I	Yes	Yes	Yes	No	Yes	n/a	n/a	Yes	Yes	No	Cannot Determine	No	Yes	No	Good
Cury-Boaventura 2018	I	Yes	Yes	Yes	No	Yes	n/a	n/a	Yes	Yes	No	Cannot Determine	No	Yes	No	Good
Cosgrove 2012	I	Yes	Yes	Yes	No	Yes	n/a	n/a	Yes	Yes	No	Cannot determine	Yes	Yes	No	Fair
Dorneles 2020	I	Yes	Yes	Yes	No	Yes	n/a	n/a	Yes	Yes	No	Cannot determine	No	Yes	Yes	Good
Duggal 2018	C	Yes	Yes	Yes	Yes	Yes	No	Yes	No	Yes	No	Cannot determine	No	n/a	No	Fair
Gustafson 2017	I/C	Yes	Yes	Yes	No	Yes	No	Yes	Yes	Yes	No	Cannot Determine	No	Yes	No	Fair
Ingram 2017	I	Yes	Yes	Yes	No	Yes	n/a	n/a	Yes	Yes	No	Cannot Determine	No	Yes	No	Good
Karim 2018	I	Yes	Yes	Yes	No	No	n/a	n/a	Yes	Yes	No	Cannot Determine	No	Yes	No	Fair
Kruger 2016	I	Yes	Yes	Yes	No	Yes	n/a	n/a	Yes	Yes	No	Cannot Determine	No	Yes	No	Good

Lavoy 2014	I	Yes	Yes	Yes	No	Yes	n/a	n/a	Yes	Yes	No	Cannot Determine	No	Yes	No	Good
Lavoy 2017	I	Yes	Yes	Yes	No	Yes	n/a	n/a	Yes	Yes	No	Cannot Determine	No	Yes	No	Good
Minuzzi 2018	I/C	Yes	Yes	Yes	No	Yes	Yes	Yes	Yes	Yes	No	Cannot determine	No	Yes	Yes	Good
Ross 2018	I/C	Yes	Yes	Yes	No	Yes	Yes	Yes	Yes	Yes	No	Cannot determine	No	Yes	Yes	Good
Schenk 2021	RCOT	Yes	Cannot determine	No	No	No	Yes	Yes	Yes	Yes	Yes	Yes	No	Yes	Yes	Good
Shimizu 2011	I	Yes	Yes	Yes	No	Yes	n/a	n/a	Yes	Yes	No	Cannot Determine	No	Yes	No	Good
Silva 2016	C	Yes	Yes	Yes	No	Yes	Yes	Yes	No	Yes	No	Cannot determine	No	n/a	Yes	Good
Simpson 2010	I/C	Yes	Yes	Yes	No	No	Yes	No	Yes	Yes	No	Cannot determine	No	Yes	No	Fair
Spielmann 2014	I/C	Yes	Yes	Yes	No	Yes	Yes	Yes	Yes	Yes	No	Cannot determine	No	Yes	Yes	Good
Spielmann 2011	C	Yes	Yes	Yes	Yes	Yes	Yes	Yes	No	Yes	No	Cannot determine	No	n/a	Yes	Good
Theall 2020	I	Yes	Yes	Yes	No	Yes	n/a	n/a	Yes	Yes	No	Yes	Yes	Yes	No	Good
Turner 2010	I	Yes	Yes	Yes	No	Yes	n/a	n/a	Yes	Yes	No	Cannot Determine	No	Yes	No	Good
Wadley 2020	I	Yes	No	Yes	No	Yes	n/a	n/a	Yes	Yes	No	Cannot Determine	No	Yes	No	Fair
Wang 2011	I	Yes	Yes	Yes	No	Yes	n/a	n/a	Yes	Yes	No	Yes	No	Yes	Yes	Good
Wong 2021	RCT	Yes	Yes	No	No	No	Yes	Yes	Yes	Yes	Yes	Yes	No	Yes	Yes	Good
vanderGeest 2017	I	Yes	No	Yes	No	Yes	n/a	n/a	Yes	Yes	No	Cannot determine	No	Yes	No	Fair
